# Αssessment of Dietary Intake and Nutritional Status of Former Opioid Users Undergoing Detoxification Process

**DOI:** 10.7759/cureus.50068

**Published:** 2023-12-06

**Authors:** Athanasios Migdanis, Ioannis Migdanis, Sousana K Papadopoulou, Laoura Hadjivasiliou, Nevena Trifonova, Maria Villioti, Constantinos Giaginis, Rena I Kosti, Odysseas Androutsos

**Affiliations:** 1 Faculty of Medicine, University of Thessaly, Larissa, GRC; 2 Department of Nutrition and Dietetics, University of Thessaly, Trikala, GRC; 3 Department of Nutrition and Dietetics, International Hellenic University, Thessaloniki, GRC; 4 Department of Food Science and Nutrition, University of the Aegean, Lemnos, GRC

**Keywords:** anthropometric characteristics, buprenorfine/naloxone, nutritional deficiencies, opioid users, nutritional status

## Abstract

Rationale: Opioid dependence is often associated with impaired nutritional status, weight changes, nutritional deficiencies, and increased sugar consumption. Scientific quantified data on the dietary habits and intake of such patients are sparse.

Methods: This was a cross-sectional study. The study was conducted among 60 male and female former heroin addicts, who sought detoxification at the OKANA replacement therapy unit, in a public university hospital. All patients were treated for their addiction with buprenorfine/naloxone in combination with counseling. With the use of an administered questionnaire, several parameters were assessed and recorded, including nutritional habits, anthropometric characteristics, recent weight and medical history, and physical activity level of the participants. Additionally, a three-day dietary recall was performed and quantified with the aid of nutritional analysis software. The results were compared with the macronutrient requirements, calculated total energy expenditure, and the population reference intake (PRI) of the participants. Finally, the level of compliance of the participants to the Mediterranean diet model was assessed using the Mediterranean diet score tool.

Results: The vast majority of the participants (77%) had a normal BMI of 18.5-25, and 15% were categorized as underweight (<18.5). Furthermore, 63% of the patients reported a mean unintended weight reduction of 9 kg over the last three months. Regarding mean energy and protein intake, no significant differences between reported intake and calculated requirements were recorded. Sugar consumption was high since it reached 20% of the total energy intake. Micronutrient intake was significantly lower for vitamins K, E, and C and potassium compared with the PRI (p=0.034, p=0.001, p=0.046, and p=0.001, respectively). Finally, a low adherence of the participants to the Mediterranean diet model was observed since 38% scored ≤15 and 62% ≤ 30 on the Mediterranean diet score tool.

Conclusions: According to the results of the study, the general nutritional status of this category of patients seems to be impaired, presenting an apparent weight reduction and an inadequate intake of some micronutrients and displaying disturbed eating behaviors. Further data on the field are required to build a future evidence base. Dietary assessment and individualized nutritional counseling, when necessary, might need to be incorporated into the typical clinical management of this patient category to avoid nutritional deficiencies and improve the withdrawal process.

## Introduction

An addiction is characterized by the compulsive need and use of drugs in spite of adverse consequences. Because the brain circuits involved in reward, stress, and self-control are functionally altered, it is considered a brain disorder [1]. It is estimated that almost 5% of the world's population currently uses drugs once a day, and nearly 0.6% suffer from a serious drug use disorder. By far, opioids, including heroin, are the most harmful types of drugs used, and cannabis is the most commonly used drug worldwide [2].

According to cohort studies on amphetamine, cocaine, and heroin users, these drugs raise the risk of morbidity, disability, and premature death. Drug use has also a wide range of negative effects on a person's psychological, emotional, and social well-being. Self-reported suicide attempts are much higher in opioid, cocaine, and amphetamine users than in non-drug users of the same age, gender, and socioeconomic status. The link is most likely mediated by depression, which is prevalent among drug users [3]. Addicts frequently have one or more co-occurring health issues, which may include lung or heart disease, stroke, cancer, or mental health problems. Infections can also be increased by drug use. HIV and hepatitis C can be transmitted through sharing injection equipment or unsafe practices such as sex without a condom. After being exposed to bacteria through injection drug use, infections of the heart and its valves and skin infections can also occur [1]. Despite addicts' general health problems, which are connected to their substance abuse, this population is also at risk of poor nutritional health, a fact that has not attracted much scientific attention so far. It has been shown by scientific studies that such people often present dysfunctional dietary patterns, weight loss, impaired nutritional status, and very often nutritional deficiencies [4,5]. Specifically, it has been observed that opioid users consume fewer meals and fewer fruits, vegetables, animal proteins, fats, and fibers than the general population [6-8]. They also have a tendency to substitute protein and fats for sugary-rich foods and alcohol, which are nutrient-deficient and, therefore, contain empty calories [6]. Micronutrient depletion is also very often present in such populations, including iron deficiency and low levels of both fat and water-soluble vitamins [9,10]. Moreover, low muscle mass and malnutrition are widespread [11].

Treatment with opioid agonists, including buprenorphine-naloxone and methadone, is the most effective pharmacotherapy for opioid addiction [12]. Disturbed eating behaviors and weight changes are also apparent during recovery from drug and alcohol addictions [13,14]. In the early phases of detoxification programs, patients often report reduced food intake due to reduced appetite, stomach disturbances, and constipation or diarrhea induced by opiate substitutes used [15]. However, in the later recovery phase, appetite and food intake seem to improve, leading to weight gain. In this period, overeating and sometimes binge eating have been reported, and the preference for foods that are high in fats and refined sugars [14,16]. High rates of eating disorders have also been observed at this period [17]. “Food addiction” and substance use seem to share a similar neurobiological and behavioral background, particularly in the way that both disrupt the parts of the brain involved in pleasure and self-control [18].

In public health, effective treatments for opioid addiction are a priority. Addiction is a serious problem that poses a significant burden on those affected. Research has reported that imbalances in certain nutrient statuses can lead to the development of barriers to withdrawal from opiate addiction [19,20]. It has also been suggested that substance abusers may have success withdrawing from opioids by following a good nutrition education program and engaging in physical activities [21]. Nutrition is connected with disorders such as diabetes mellitus and vitamin D deficiency, which can have an effect on morphine dependence [22]. Moreover, nutritional status can be crucial to the recovery and survival of addicts who might suffer from infections such as HIV, which poses a threat to nutritional status and may result in malnutrition [23].

The assessment of nutritional status and, by extension, the implementation of effective nutritional intervention in ex-opioid users during detoxification could reduce their nutritional deficiencies and improve their glycemic profile and general nutritional status, as well as the outcome of their path to withdrawing from opioids. Scientific quantified data on the dietary intake (energy and micro-macronutrients) of such patients are sparse. The aim of the present study was to assess the nutritional intake of former opioid addicts who were in the detoxification process. Other parameters including anthropometric characteristics, nutritional habits, and the level of compliance with the Mediterranean diet model were also assessed.

## Materials and methods

This was a cross-sectional study. The study was initiated in November 2022 and completed in June 2023. The study was conducted among 60 male and female former heroin addicts, who sought detoxification at the OKANA (Organization Against Drugs) replacement therapy unit, in a public university hospital (General University Hospital of Larissa, Greece). All patients were treated for their addiction with buprenorfine/naloxone in combination with counseling. Patients who had a chronic or acute condition (e.g., inflammatory bowel disease, food allergies, had undergone major surgery, or had a recent infection) that might have affected their recent nutritional status were excluded from the study. The trial protocol was approved by the Hospital's Ethics Committee, and adhered at all times to the Helsinki Declaration. An administered questionnaire was used to assess and record several parameters, including anthropometric characteristics (weight, height, BMI), level of education, recent medical and weight history, physical activity level (intensity, duration, and type of physical activity per day), and nutritional habits/patterns of the participants. Moreover, a three-day dietary recall was carried out and quantified with the assistance of nutritional analysis software. 

In order to gather the data, participants were asked to accurately recall all meals and beverages ingested over the last three non-consecutive days: two days during the week and one weekend day (a non-typical day), using the three-day dietary recall approach [24,25]. Subjects provided descriptions concerning cooking techniques, ingredients, food preparation, and portion sizes. A registered dietitian collected the data. All relevant dietary supplements were also noted and incorporated into the data analysis. The acquired data were analyzed with the aid of nutritional analysis software (dietSpeak) that included the USDA food composition database. In order to conduct our analysis of the collected data, the mean macronutrient and micronutrient intake values over the course of three days were used. Informed consent was obtained from all patients before entering the study.

The results were compared with the calculated total energy expenditure using the Mifflin-St Jeor equation [26], macronutrient requirements, and the population reference intakes (PRIs) of the participants according to the European Food Safety Authority (EFSA) [27]. Finally, the level of compliance of the participants to the Mediterranean diet model was assessed using the Mediterranean diet score tool (MedDiet score) [28]. The MedDiet score is a tool created to assess the adherence of a population to the Mediterranean diet, which worldwide has been recognized as a model for a healthy balanced diet. The main components that comprise this dietary pattern are vegetables, legumes, fruits and nuts, unrefined cereals, and olive oil. Another essential component is the low consumption of saturated fats coming from animal sources [29]. The MedDiet score tool specifically uses 11 main components of the Mediterranean diet (legumes, olive oil, poultry, red meat, non-refined cereals, vegetables, fruits, fish, potatoes, dairy products, and alcohol). To assess the level of consumption of the above food groups, a 5-point scale is used, ranging from 0=no consumption to 5=daily consumption, respectively.

Statistical analysis

Statistical analysis was conducted using Statistical Product and Service Solutions, version 26 (IBM SPSS Statistics for Windows, Armonk, NY). The Kolmogorov-Smirnov test was carried out to determine the normality of the distribution of the examined variables. Continuous variables such as age, anthropometric characteristics, macro-micronutrient intake, and MedDiet score are presented as means and ± standard deviations. A comparison of continuous variables (energy and protein intake) and calculated requirements was carried out using the paired samples t-test. To assess possible differences between micronutrient intake and PRIs, a one-sample t-test was performed. To examine the possible relation between adherence to the Mediterranean diet and weight changes, one-way ANOVA was used. Statistical significance is reported as p<0.05.

## Results

A total of 60 patients consented and were recruited for the study. The mean age of the participants was 37.4±8 years (63% males and 37% females). The vast majority of the participants (77%) had a normal BMI of 18.5-25, and 15% were categorized as underweight (<18.5). Furthermore, 63% of the patients reported a mean unintended weight reduction of 9±5.3 kg over the last three months (Table 1).

**Table 1:** Demographic and anthropometric characteristics and nutritional intake of the participants BMI, body mass index; SD, standard deviation

	mean	minimum	maximum	SD
Age (years)	37.4	23	63	8.1
Weight (kg)	65.8	45	87	9.9
BMI (kg/m^2^)	21.3	16	28	2.7
Energy intake (kcal/day)	2039.3	819	3925	798.6
Protein intake (gr)	74.5	18	201	48.5
Iron (mg/day)	18.3	1	113	22.6
Vit K (μg/day)	62.9	18	97	21.4
Vit E (mg/day)	4.1	0	25	4.5
Vit C (mg/day)	75.7	3	366	82.4
Cho (g/day)	250.4	62	597	122
Fat (g/day)	99	16	271	53
Potassium (mg/day)	2149	187	6219	1366.4
MedDiet Score	17.2	5	25	3.9
Sugars (g/day)	96.1	4	262	64.8
Fat (g/day)	28.8	3	119	24.7
Fibre (g/day)	13.9	1	42	10
Physical activity (min/day)	25.8	0	180	38.8
Increase in weight (kg) over last 3 months (n=9)	7.3	2	30	9.7
Decrease in weight (kg) over last 3 months (n=38)	9.3	1	30	5.3

**Figure 1:**
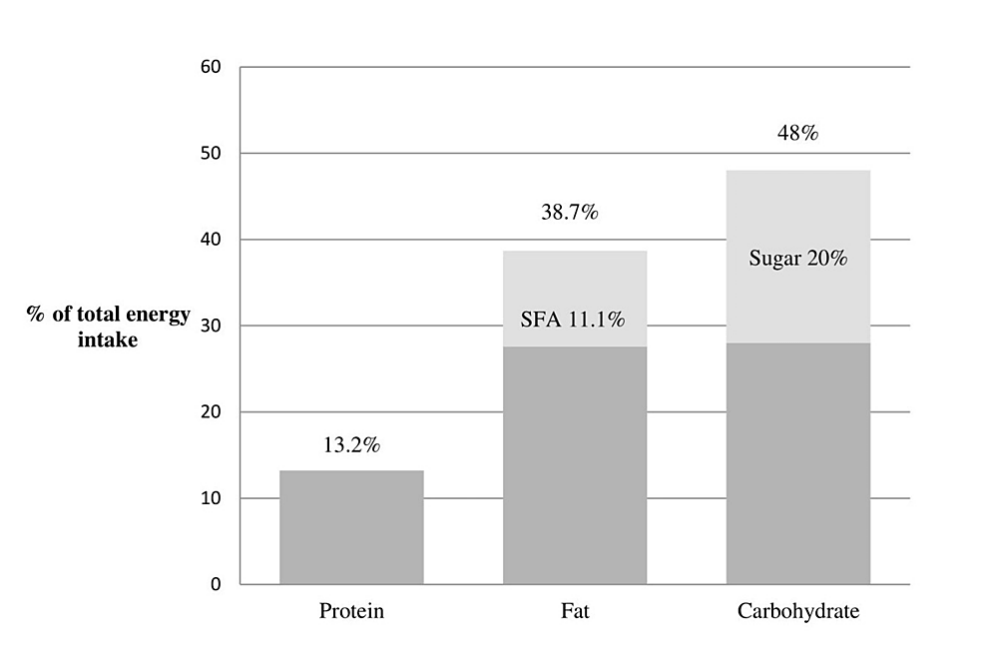
Macronutrient contribution to the total energy intake SFA, saturated fatty acids

Regarding mean energy and protein intake, no significant differences between reported intake and calculated requirements were recorded (Table 2). The mean energy intake of the participants was 2,039.3±798.6 kcal/day, which was lower than the calculated requirements of 2,166.8±229 kcal/day, but not significantly lower. As for the macronutrient intake, 13.2% of the total energy intake was derived from protein, 38.7% from fat, and 48% from carbohydrates. Sugar consumption was high since it reached 20% of the total energy intake (Figure 1). Concerning dietary fiber, the mean intake of the participants was found to be significantly lower than the PRI proposed value (p=0.001) (Table 2).

**Table 2:** Differences between dietary intake and requirements of the participants SD, standard deviation; AI, adequate intake; PRI, population reference intake

Macro/micronutrients	Daily intake (mean ± SD)	Calculated requirements (mean ± SD)/AIs & PRIs	p value
Energy intake (kcal/day)	2039.3±798.6	2166.8±228.8	0.217
Protein intake (gr/day)	74.5±48.5	65.8±9.9	0.123
Vit K (μg/day)	62.9±21.4	70	0.034
Iron (mg/day)	18.3±22.6	11-16	0.428
Vit E (mg/day)	4.1±1.5	11-13	0.001
Vit C (mg/day)	75.7±22.4	95-110	0.046
Dietary fibre (g/day)	13.9±10	25	0.001
Potassium (mg/day)	2149±1366	3500	0.001

Micronutrient mean intake was significantly lower for vitamins K, E, and C and potassium compared with the PRIs (p=0.034, p=0.001, p=0.046, and p=0.001, respectively) (Table 2). Finally, a low adherence of the participants to the Mediterranean diet model was observed since 38% scored ≤15 and 62% ≤30 on the MedDiet score tool. It was observed that, from the analysis conducted, participants who had an unintended reduction in their body weight over the last three months had a significantly lower adherence to the Mediterranean diet, compared to the groups who did not experience any weight loss or had an increase in their weight (p=0.045) (Table 3).

**Table 3:** Relation between adherence to the Mediterranean diet and weight changes SD, standard deviation

	Increase in weight (last 3 months) (n=9)	No change in weight (last 3 months) (n=13)	Decrease in weight (last 3 months) (n=38)	p value
MedDiet Score (mean±SD)	19±3.4	18±4	16.4±4	0.045

## Discussion

The study’s findings indicate that opioid addiction can have a negative impact on the overall nutritional status of this category of patients, which is associated with changes in anthropometric characteristics, insufficient intake of some micronutrients, and irregular eating patterns. In the present study, the majority of the participants had a normal BMI, while 15% of them had a BMI of below 18.5 kg/m^2^. These findings seem to be in agreement with similar studies that have assessed the anthropometric characteristics of such patients [5,19,30]. In a study by Saeland et al., which was conducted on 195 drug addicts living in Oslo, Norway, 10% of the women and 3% of the men were found to have a BMI below the normal range [30]. In another study with a cross-sectional design [19], the authors addressed the nutritional status of 49 heroin addicts in methadone maintenance treatment. The results showed that again 10.2% of the participants had a BMI below 18.5 kg/m^2^. In another study carried out on opioid addicts who were also in the detoxification process via methadone-assisted treatment, authors reported a percentage of underweight BMI of 9% [5]. It was observed in the present study that 63% of the patients reported a mean unintended weight reduction of 9 kg over the last three months. It has been described in the international literature that, generally, substance use can have an impact on the user’s nutritional status [31]. The lifestyle of this population tends to be chaotic and disorganized, and money is typically spent on drugs rather than food. It has been shown that opiates' short-term effects include anorexia, decreased food intake, and impaired gastrointestinal motility, all of which eventually can result in malnutrition and a compromised nutritional status [32]. Additionally, it needs to be mentioned that patients who receive pharmacotherapy during the early stages of detoxification often report a period of low food intake, during which eating becomes their least important task due to nausea, anorexia, and gastrointestinal abnormalities, all of which can make eating challenging [33].

As far as the energy intake of such patients is concerned, the majority of the literature focuses mainly on nutritional status and dietary habits/patterns, but less has been published on quantified energy intake per day. In the present study, the mean energy intake of the participants was 2,039±798.6 kcal/day, which was below the calculated requirements, without the difference reaching statistical significance. According to the information acquired from the participants, most reported that, during the early stages of the detoxification process, food was low down their priorities list mainly due to nausea and stomach upsets and intermittent constipation and diarrhea. In a similar study where the researchers assessed the dietary intake of heroin addicts in methadone maintenance treatment via a food frequency questionnaire, the results showed that mean daily intake was at 2,325 kcal/day, although the energy requirements of the participants were not calculated in order to see a comparison [19]. In the study from Norway where a 24-hour recall was used to assess, among others, the energy intake of drug addicts, it was seen that males consumed a mean energy intake of 2,199 kcal/day and females 1,625 kcal/day [30]. Again, unfortunately, the energy requirements of the study sample were not calculated, since it would be interesting to see if they were met or not.

With regards to macronutrient percentages of total energy intake, the analysis showed 13.2% coming from protein, 38.7% from fat, and 48% from carbohydrates. Sugar consumption was high since it reached 20% of the total energy intake. According to the existing literature and previous studies that have concentrated on the subject, it is supported that people addicted to opiates have increased preferences for sweet foods [6,15,34]. This may be due to the pharmacological effect of heroin on specific brain receptors. Sugar is known to increase endogenous opioids, and heroin is known to depress the production of these same endogenous opioids. Craving for sugar may be a physiological response to reduced endogenous opioids caused by heroin [33]. It has been reported that, between the first and sixth month of detoxification, a high preference and craving for table sugar and sweet foods, such as cakes and confectionary foods, often takes place as a replacement for the drug [35]. Studies have also revealed a prolonged period of abstinence from drugs when excessive amounts of sugar are consumed [7]. It seems that studies that have tried to address the matter have shown relative results. In the study conducted in Norway [30], total carbohydrates accounted for 60% of total energy intake and simple sugars reached as much as 30% of the total energy intake. It was also obtained, from the questionnaires used in the study, that sugars and sugar-sweetened food items were preferred by 61% of the respondents, which was mirrored in their biochemical markers as 12% of the males and 20% of the females had concentrations of HbA1c above the reference value. Other studies that focused on addicts being on methadone maintenance treatment revealed also high percentages of total energy intake coming from sugars that ranged from 18.5% to 30.5% [7,19].

Another parameter that was also attempted to be examined in the present study was the micronutrient intake of the participants. Generally, not many studies in the literature have concentrated on assessing the micronutrient intake of such a population. In a study from Oslo [30], the intake of the majority of vitamins and minerals (iron, calcium, vitamins D and C, magnesium, selenium, and some B vitamins) was below the recommended intake according to the Nordic Nutrition Recommendations (NNR). This was mainly attributed to the fact that most participants had limited access to nutrient-dense high-quality food options. Other studies that measured plasma levels of micronutrients, instead of nutritional intake, reported low selenium, potassium, iron, and vitamins A, C, D, and E in both alcohol and drug addicts being on treatment or not [4,36,37]. In the present study, the analysis revealed vitamins K, E, and C and potassium intake below the recommended values according to the European Food Safety Authority (EFSA) PRIs [27]. Thus, it seems that the incorporation of vitamin and mineral supplementation into detoxification programs requires consideration.

Furthermore, in an effort to understand the nutritional behaviors/habits of such a group of patients, the level of compliance of the participants to the Mediterranean diet model was assessed using the Mediterranean diet score tool (MedDiet score). MedDiet is a plant-based diet comprising a high amount of vegetables, fruit, cereal, nuts, and extra virgin olive oil; a moderate consumption of fish, poultry, and dairy products; and a low intake of processed red meat products and saturated fats [29]. The Mediterranean diet has been recognized as a balanced beneficial nutritional pattern that decreases the risks of a variety of human disorders and pathological states and promotes human health [38]. Generally, little research is available examining the food preferences of either active substance users or those undergoing different detoxification processes. Socioeconomic factors, such as education, income, presence of family or partner, food preparation skills, and living conditions, seem to be related and can have an impact on the nutritional behaviors of such individuals [35]. As mentioned before, it has been seen that people addicted to opiates typically consume meals high in refined carbohydrates and fat and low in fruit and vegetables. Moreover, very often they have irregular eating patterns, taking only one or two meals per day and staying without food for longer periods of time [6,33]. The way of living of this group of people is usually disrupted, and money is preferably spent on the support of their habits, rather than on quality food. The nutritional imbalance observed in such patients is also correlated to other parameters such as their knowledge of nutrition and the presence of accompanying infectious diseases such as HIV and hepatitis that are common in heroin users. In studies where subjects received information on balanced nutrition as part of a treatment program, significantly healthier eating habits were achieved, and more meals were consumed [34,39]. Moreover, it has been noted by research that HIV-positive substance abusers have more energy, fat, and protein-deficient diets compared with substance abusers who are HIV-negative [40]. In the present study, the results revealed a low adherence of the participants to the Mediterranean diet, and interestingly weight loss was related to the extent to which participants actually followed a balanced diet since those who had an unintended reduction in their body weight over the last three months had a significantly lower adherence to the Mediterranean diet compared to the groups who did not experience any weight loss or had an increase in their weight. Limitations of the study include restricted sample size and a single-center data collection, which can potentially affect the results’ external validity. However, our findings can suggest directions for future research with larger sample sizes and a multicenter design. Finally, another limitation could be considered the method used to assess the nutritional intake of the participants (three-day dietary recall), which, despite its many advantages, is sometimes associated with over- or underreporting [24].

## Conclusions

We conclude, according to the results of the study, that the general nutritional status of this category of patients seems to be impaired, presenting an apparent weight reduction, an inadequate intake of some micronutrients, increased sugar consumption, and displaying disturbed eating behaviors with low adherence to the Mediterranean diet. Further data on the field are required to build a future evidence base. Future studies could focus on assessing the body composition of such patients (e.g., bioelectrical impedance analysis) and the daily intake of a broader range of micronutrients. Nutritional assessment and individualized nutritional counseling, when required, might need to be included in the routine clinical management of this patient category to avoid nutritional deficiencies and malnutrition, disturbed glycaemic control, and improve the withdrawal process.
